# DLSIA: Deep Learning for Scientific Image Analysis

**DOI:** 10.1107/S1600576724001390

**Published:** 2024-03-21

**Authors:** Eric J. Roberts, Tanny Chavez, Alexander Hexemer, Petrus H. Zwart

**Affiliations:** a Center for Advanced Mathematics for Energy Research Applications, Lawrence Berkeley National Laboratory, Berkeley, CA 94720, USA; b Molecular Biophysics and Integrated Bioimaging Division, Lawrence Berkeley National Laboratory, Berkeley, CA 94720, USA; c Advanced Light Source, Lawrence Berkeley National Laboratory, Berkeley, CA 94720, USA; d Berkeley Synchrotron Infrared Structural Biology Program, Lawrence Berkeley National Laboratory, Berkeley, CA 94720, USA; DESY, Hamburg, Germany

**Keywords:** deep learning, convolutional neural networks, X-ray scattering, tomography, data compression

## Abstract

DLSIA (Deep Learning for Scientific Image Analysis) is a Python-based machine learning library that empowers scientists and researchers across diverse scientific domains with a range of customizable convolutional neural network (CNN) architectures for a wide variety of tasks in image analysis to be used in downstream data processing. DLSIA features easy-to-use architectures, such as autoencoders, tunable U-Nets and parameter-lean mixed-scale dense networks (MSDNets). Additionally, this article introduces sparse mixed-scale networks (SMSNets), generated using random graphs, sparse connections and dilated convolutions connecting different length scales. For verification, several DLSIA-instantiated networks and training scripts are employed in multiple applications, including inpainting for X-ray scattering data using U-Nets and MSDNets, segmenting 3D fibers in X-ray tomographic reconstructions of concrete using an ensemble of SMSNets, and leveraging autoencoder latent spaces for data compression and clustering. As experimental data continue to grow in scale and complexity, DLSIA provides accessible CNN construction and abstracts CNN complexities, allowing scientists to tailor their machine learning approaches, accelerate discoveries, foster interdisciplinary collaboration and advance research in scientific image analysis.

## Abstract 2

This article introduces DLSIA (Deep Learning for Scientific Image Analysis), a user-friendly Python library that provides scientists and researchers with a number of convolutional neural networks with customizable architectures for a variety of image analysis tasks, enabling accelerated discoveries and enhancing interdisciplinary collaboration in the face of growing experimental data complexity.

## 1. Introduction

### 1.1. Purpose and motivation

Scientific image analysis forms a crucial component of numerous workflows at user facilities, generating an abundance of data sets that each possess unique characteristics. Given the distinct nature of these data sets, the need frequently arises to craft custom solutions tailored to individual experiments. Convolutional neural networks (CNNs), along with other machine learning tools, prove extremely valuable in this regard, capable of addressing a variety of analysis needs and producing insightful results. The unique aspect of scientific data analysis in such settings often necessitates the creation of bespoke solutions tailored to individual experiments, providing optimal results given the data’s specific characteristics. CNNs, along with a host of other machine learning tools, present themselves as exceptionally suitable for such tasks because of their flexibility and the wide array of potential applications to which they cater.

### 1.2. Background and prior art

CNNs have emerged as a transformative class of machine learning models specifically designed to unravel patterns and extract meaningful features from various forms of data. Having gained significant popularity in the scientific community, CNNs are particularly well suited for tackling image analysis tasks, including object detection, image classification and pixel-by-pixel semantic segmentation. The unique strength of CNNs lies in their ability to autonomously learn discriminative features directly from the data themselves, eliminating the need for laborious manual feature engineering. By training on large data sets with labeled examples, CNNs can learn to recognize specific objects, identify anomalies or detect subtle patterns. Moreover, CNNs remain a versatile tool, allowing researchers from different backgrounds to choose from a variety of different CNN architectures that can denoise, reconstruct and segment images (Xing *et al.*, 2017 ▸; Kaur *et al.*, 2018 ▸; Manifold *et al.*, 2019 ▸; Gong *et al.*, 2019 ▸; Kromp *et al.*, 2020 ▸; Jung & Kim, 2014 ▸), or perform higher-level tasks from among their diverse scientific disciplines, including automated structure and material classification and data-driven discovery in X-ray scattering (Kiapour *et al.*, 2014 ▸; Liu *et al.*, 2019 ▸; Deyhle *et al.*, 2018 ▸; Douarre *et al.*, 2018 ▸; Wang *et al.*, 2017 ▸), biological (Radivojević *et al.*, 2020 ▸; Wäldchen & Mäder, 2018 ▸), crystallographic (Ziletti *et al.*, 2018 ▸; Kirman *et al.*, 2020 ▸; Sun *et al.*, 2019 ▸) and signal processing (Tabar & Halici, 2016 ▸; Schirrmeister *et al.*, 2017 ▸; Lawhern *et al.*, 2018 ▸; LiKamWa *et al.*, 2016 ▸) settings.

While the widespread adaptability of CNNs has made them a prevalent tool across various scientific domains, not all scientific researchers possess the expertise or knowledge required to construct and train these networks effectively. Access to user-friendly libraries with pre-built networks is invaluable for individuals lacking a deep understanding of CNNs. These libraries offer a convenient way to deploy CNNs without dealing with network architecture intricacies. Researchers can focus on their domain expertise by leveraging these libraries instead of building CNNs from scratch. The flexibility of these libraries enables iterative experimentation, allowing researchers to easily swap network architectures and adjust hyperparameters to find the best configurations for their problems. Access to state-of-the-art networks saves time and resources, while promoting interdisciplinary collaboration by abstracting the complexities of CNN construction and training, as researchers can focus on their areas of expertise while leveraging the power of CNNs for their analyses.

In summary, the prevalence of CNNs in the sciences necessitates user-friendly libraries that simplify their construction and training, allowing scientists to stay at the forefront of CNN research without the need for extensive expertise in deep learning. To address these challenges and expedite the process of incorporating machine learning into scientific image analysis workflows, we introduce DLSIA (Deep Learning for Scientific Image Analysis), a Python-based general-purpose machine learning library offering a flexible and customizable environment for generating custom CNN architectures and an extensive suite of tools designed to empower scientists and researchers from diverse scientific domains, including beamline scientists, biologists and researchers in X-ray scattering. DLSIA enables a seamless integration of custom CNN architectures and other advanced machine learning methods into common workflows, providing researchers with the means to rapidly test and implement different analysis approaches within a unified framework and dramatically increasing efficiency and adaptability. Whether the task at hand involves image classification, anomaly detection or any other complex pattern recognition, DLSIA offers a streamlined, efficient platform that enables users to explore, compare and customize a wide array of CNN architectures, facilitating a systematic investigation of what works, what does not work and what is best suited for their specific scientific problems.

### 1.3. The DLSIA software library

The core focus of DLSIA lies in its ability to bridge the gap between cutting-edge deep learning techniques and the challenges encountered in scientific image analysis. By offering a comprehensive collection of user-customizable CNNs, including autoencoders, tunable U-Nets, mixed-scale dense networks and more novel randomized sparse mixed-scale networks, DLSIA allows researchers to harness the power of state-of-the-art deep learning while tailoring a network architecture to the specific demands of their scientific investigations. This flexibility empowers users to fine-tune CNNs, select appropriate layers, optimize hyperparameters and explore diverse architectural variations, enabling a comprehensive exploration of the rich design space inherent in deep learning based image analysis.

DLSIA facilitates seamless integration with various scientific data sets and promotes reproducible research through its intuitive and extensible PyTorch application programming interface (API). It offers a rich set of functionalities for data preprocessing, model training, validation and evaluation, while also providing convenient visualization tools to aid in the interpretation and analysis of results. With its user-centric design philosophy, DLSIA aims to empower scientists across domains to leverage the potential of CNNs for scientific image analysis, ultimately accelerating discoveries and advancing research in a wide range of scientific fields. DLSIA documentation and core may be accessed at https://dlsia.readthedocs.io/en/latest/, while a list of DLSIA modules, scripts and subroutines is given in Appendix *A* [App appa].

The rest of the article is organized as follows: Section 2 takes an in-depth look at the CNN architectures offered; Section 3 describes the different utility functions, data loaders, training regiments and uncertainty quantification available to DLSIA users; we validate DLSIA CNN architectures through various applications on experimental data in Section 4 and offer insights regarding network selection and initializing hyperparameter tuning; and Section 5 concludes with a discussion of DLSIA results and viability.

## 2. DLSIA deep convolutional neural networks

CNNs are deep learning models that excel at visual data analysis. In general, CNNs capture features by applying many convolutional filters, or kernels, to local regions of the data via several adjacently connected convolutional layers. The filters are square matrices with adjustable weights that serve as ‘windows’ observing a specific region of the image. By learning the filters’ weights via network training and optimization, CNNs can identify various features within the image.

We highlight below the different CNN architectures available in the DLSIA software library. Each available network varies in its sequencing of layers and addition of nonlinear activation, pooling and normalization layers to decompose images into complex hierarchical structures and increase the expressive power. But true to the original goal of DLSIA, all networks are fully customizable with an array of user-specified hyperparameters available to toggle.

### 2.1. Tunable U-Nets

Included in the DLSIA software suite is a tunable variant of U-Nets (TUNets), a popular and effective deep CNN (Ronneberger *et al.*, 2015 ▸). Inspired by autoencoders (Section 2.2) and first introduced for the segmentation of biomedical images, its distinctive U-shaped architecture consists of typically mirrored contractive encoder and expansive decoder halves. Contextual information and features are captured by the contractual encoder phase, made up of a predefined number of layers *d*, each consisting of stacked unpadded convolutional operators. Max-pooling operations between layers reduce the spatial dimensionality to ease computational costs, introduce translational equivariance (Finzi *et al.*, 2020 ▸) and encourage higher-level feature extraction. Next, the expansive decoder half mirrors the downsampling phase, but with transposed convolutions between layers to recover the previously compressed spatial dimensions, effectively projecting the encoder’s learned features into the higher resolutions of the original image space to predict a pixel-by-pixel semantic segmentation (Noh *et al.*, 2019 ▸; Springenberg *et al.*, 2014 ▸). Moreover, long-reaching skip connections are introduced in the form of channel-wise concatenations of intermediate feature maps between adjacent contractive and expansive phases, encouraging an aggregation of multi-scale feature representation at different network stages (Zhou *et al.*, 2018 ▸, 2020 ▸; Kumar *et al.*, 2018 ▸; Drozdal *et al.*, 2016 ▸).

TUNet performance on different applications relies significantly on the various hyperparameters that govern the network architecture (Kinnison *et al.*, 2018 ▸; Li *et al.*, 2021 ▸; Berral *et al.*, 2021 ▸). As such, the DLSIA API offers full flexibility in creating and deploying TUNets of custom sizes and morphology by allowing the user to define the four following architecture-governing hyperparameters:

(i) Depth *d*: the number of layers in the TUNet. A depth of *d* will contain *d* layers of dual convolutions and accompanying intralayer operations in each of the encoder and decoder phases, with *d* − 1 mirrored max-pooling, up-convolutions and concatenation steps between each layer.

(ii) Number of initial base channels *c*_b_. The input data are mapped to this number of feature channels after the initial convolution.

(iii) Growth rate *r*: the growth rate/decay rate of feature channels between successive layers.

(iv) Hidden rate *r*_h_: the growth rate/decay rate of feature channels within each individual layer, between each layer’s successive convolutions.

**Figure 1:**
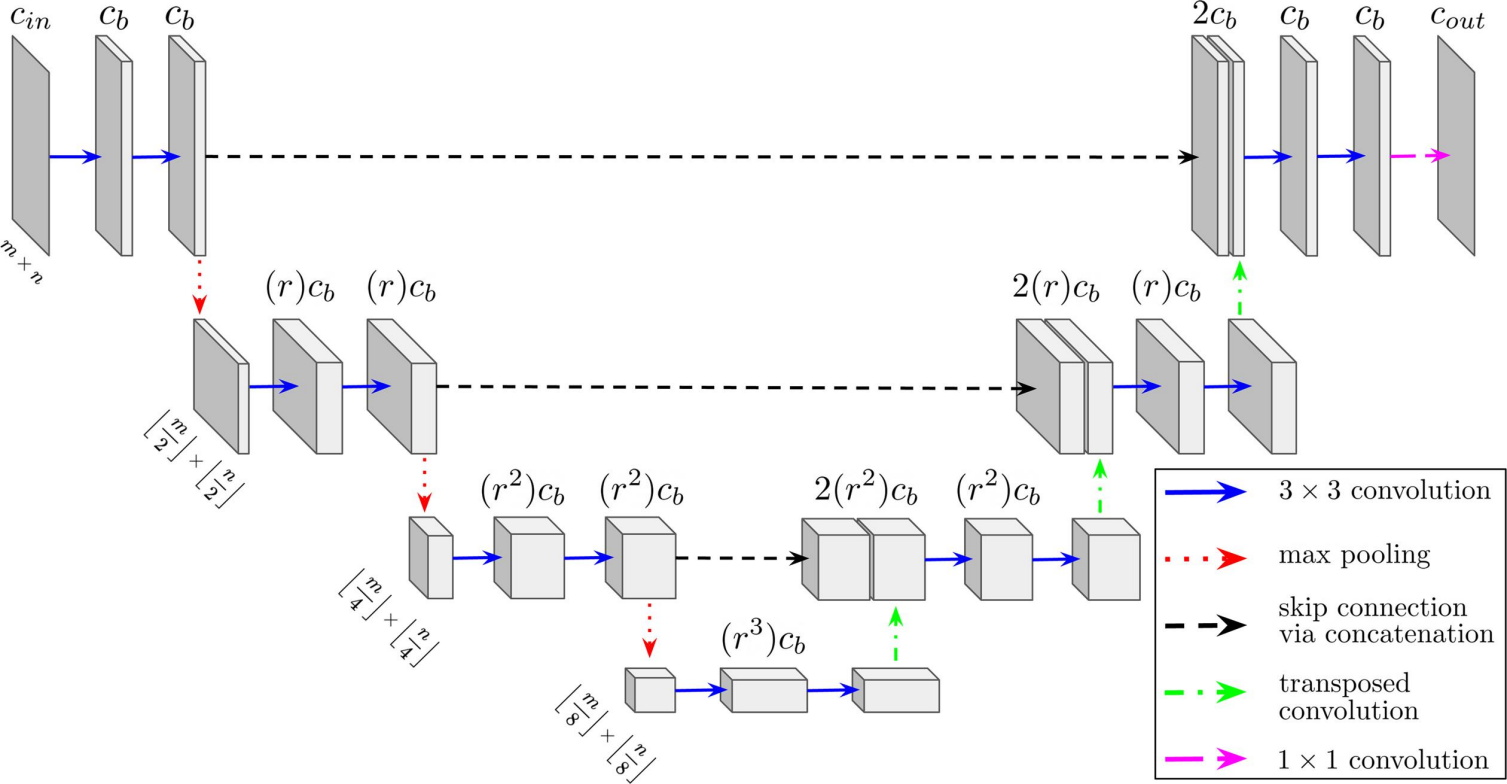
Diagram of a 2D four-layer tunable U-Net congruent with input data of *c*_in_ channels and spatial dimensions *m* and *n*. Among the user-defined hyperparameters on display are the initial base channels *c*_b_ and the channel growth factor *r*, both of which control the size of the network and thus its potential expressive power. The hidden growth rate *r*_h_ is set to 1 for simplicity. We note that DLSIA easily accommodates volumetric data by simply replacing all convolutions (and associated layer normalization) with their 3D counterparts.

The original implementation of U-Nets (Ronneberger *et al.*, 2015 ▸) uses the following default hyperparameters which may be used as a starting point for finding an appropriate architecture for specific applications: *d* = 4, *c*_b_ = 64 and *r* = 2, where *r*_h_ is exclusive to DLSIA, is typically set to 1, but can be toggled for model fine-tuning purposes along with *c*_b_ and *r*. Additionally, DLSIA defaults to rectified linear unit (ReLU) nonlinear activation and batch normalization after each convolution operation to expedite the learning process (Ioffe & Szegedy, 2015 ▸). A U-Net schematic of depth *d* = 4 is shown in Fig. 1 ▸, depicting the order of operations and evolution of channels and spatial dimensions along the contracting and expanding halves. We note that the growth and hidden rates of feature channel growth and decay may be non-integers.

### 2.2. Convolutional autoencoder

**Figure 2:**
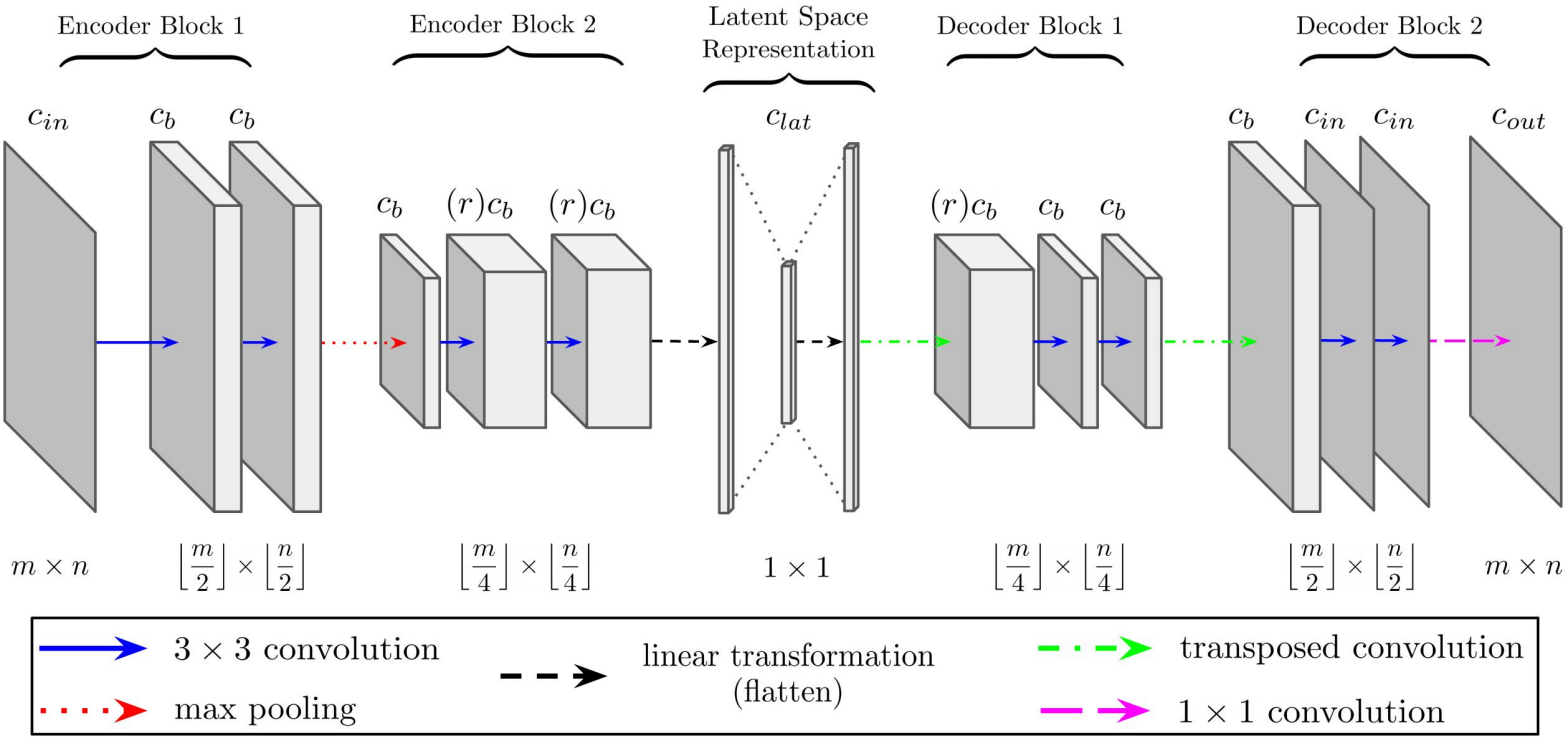
Schematic overview of a two-layer autoencoder congruent with input data of *c*_in_ channels and spatial dimensions *m* and *n*. DLSIA provides the flexibility to adjust the following hyperparameters: initial base channels *c*_b_, channel growth factor *r* and length of latent space vector *c*_lat_.

Convolutional autoencoders are a deep unsupervised neural network framework generally tasked with learning feature extraction for the purpose of reconstructing the input (Rumelhart *et al.*, 1985 ▸; LeCun *et al.*, 1998 ▸). While relatively simple in structure and acting as a precursor to U-Net encoder–decoder structure, the difference displayed in Fig. 2 ▸ shows the encoder half terminating at a single-dimensional latent space of features, often referred to as the latent space representation. This informational ‘bottleneck’ forces the network to learn only the most important features and contextual information. The second half of the network, the decoder, concludes with alternating transposed convolutions and blocks of dual convolutions to project the information back to the input space and learn the reconstruction of input data.

DLSIA instantiation of autoencoders once again reflects that of the tunable U-Nets. Users may find the autoencoder with the appropriate expressive power to suit their needs by toggling the number of layers *d*, the initial number of base channels *c*_b_ and the growth rate *r* of the convolutional channels. Additionally, users are encouraged to experiment with different sizes of latent space vectors with the *c*_lat_ hyperparameter, as an appropriate *c*_lat_ may vary by several orders of magnitude depending on the size and scope of the given application.

### 2.3. Mixed-scale dense CNNs

**Figure 3:**
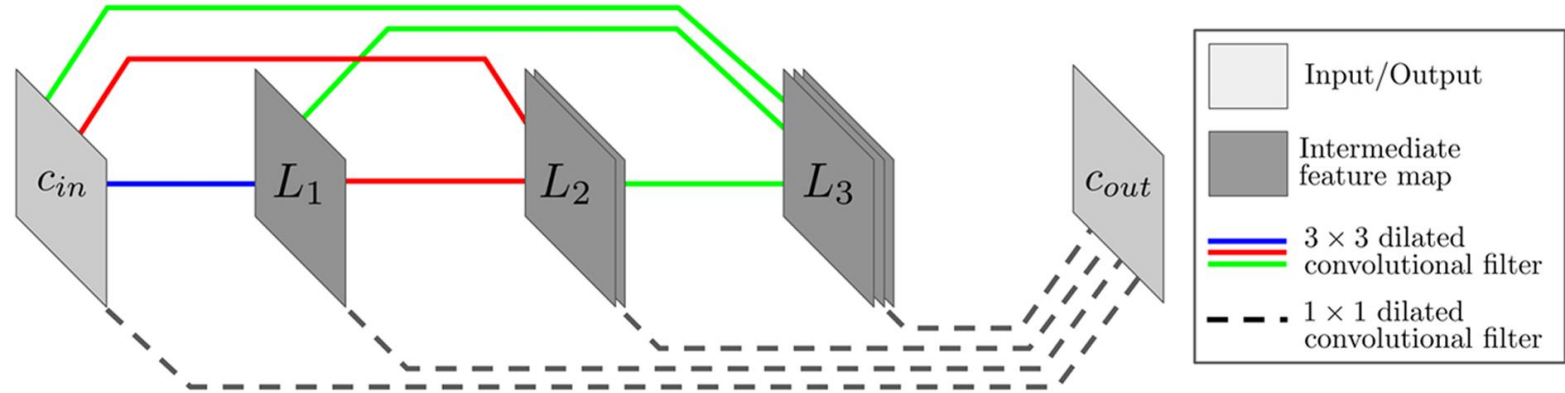
Schematic of a three-layer MSDNet with *c*_in_ and *c*_out_ the number of input and output channels. Blue, green and red solid lines represent 3 × 3 dilated convolutions between all possible pairs of input and intermediate layers, with different dilations assigned to each color. The black dashed lines at the bottom connecting all input and intermediate layers to the output layer represent 1 × 1 convolutional operators, amounting to a linear sum between individual pixels at each position among all non-output layers.

The MSDNet was developed as a deep learning framework with a relatively simple architecture containing approximately two to three orders of magnitude fewer trainable parameters (Pelt & Sethian, 2018 ▸; Pelt *et al.*, 2018 ▸) than U-Nets and typical encoder–decoder networks. MSDNets reduce model complexity in two ways. First, to probe image features at different length scales and preserve dimensionality between all network layers, dilated convolutions (Yu & Koltun, 2015 ▸) replace the upscaling and downscaling operations typically found in CNNs. Convolutions of integer dilation *l* consist of the same square kernel as their non-dilated counterparts, though the dilated kernel’s receptive field is expanded by spacing neighboring entries (*l* − 1) pixels apart in horizontal and vertical directions. Secondly, as depicted in the three-layer MSDNet diagram in Fig. 3 ▸, layers associated with different length scales are mixed together by densely connecting all potential pairs of layers, leading to several advantages, including maximum feature reusability, recovery of spatial information lost in the early layers and alleviation of the vanishing gradient problem (Ioffe & Szegedy, 2015 ▸) that plagues deep networks (Tong *et al.*, 2017 ▸). The final MSDNet output layer is computed by replacing dilated convolutions with 1 × 1 non-dilated convolutions. These single-pixel filters connecting all layers result in a linear combination of intermediate feature maps with weights learned during the optimization process.

Overall, MSDNets have a much simpler architecture than the aforementioned U-Net design. As a result, the DLSIA API requires only two main hyperparameters with which to govern the network architecture.

(i) Depth *d*: the number of network layers.

(ii) Maximum dilation *l*_m_: the maximum integer dilation of the network, where either

(*a*) each layer *d*_*i*_ is assigned integer dilation 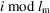 , or

(*b*) DLSIA users can manually assign specific (custom) dilations to each layer with a vector of length *d*, *e.g.* cycling through dilations of size [1, 2, 4, 8, 16] ten times in a network with *d* = 50.

The original implementation of MSDNets used *d* = 100 and *l*_m_ = 10 for all applications (Pelt & Sethian, 2018 ▸), though we encourage users to experiment with larger dilation sizes, either with *l*_m_ > 10 or manually specifying powers of 2 such as [2^0^, 2^1^, 2^2^,…, 2^*n*^].

### 2.4. Sparse mixed-scale CNNs

MSDNets are designed to require a minimal number of parameters, yet the resulting networks may still be trimmed down using pruning approaches. For instance, results from the graph-based pruning method LEAN (Schoonhoven *et al.*, 2020 ▸) demonstrate that large MSDNets can be reduced to 0.5% of their original size without sacrificing significant performance. Given the high quality in performance of pruned networks in general (Blalock *et al.*, 2020 ▸; Park *et al.*, 2016 ▸; Wang *et al.*, 2021 ▸), it would be advantageous to be able to create pre-pruned networks from scratch, aimed at producing networks that are as lean as possible with the lowest chances of overfitting.

**Figure 4:**
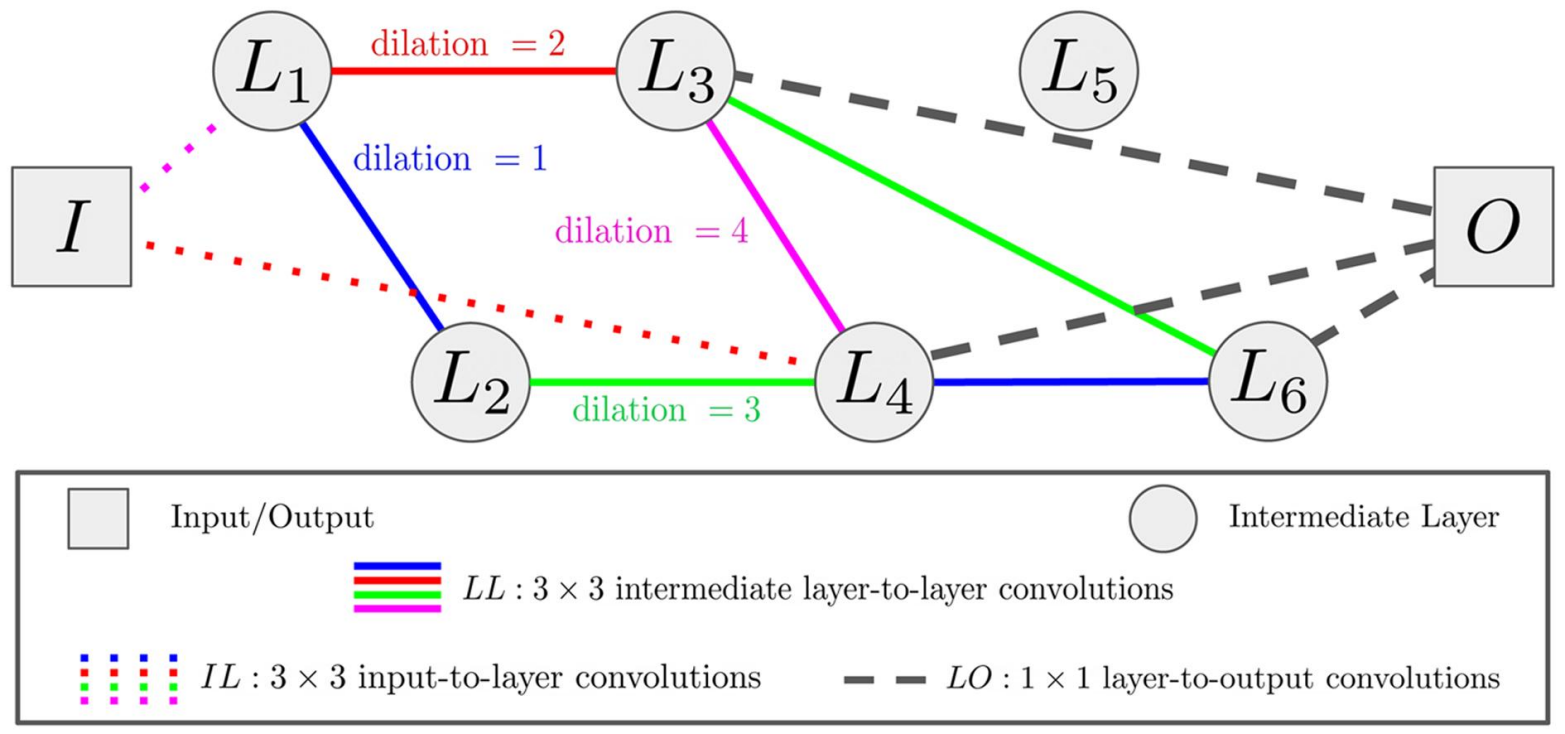
Schematic overview of a six-layer SMSNet. Network nodes consist of the input data *I*, six intermediate (hidden) layers *L* and output data *O*. All nodes/layers are sparsely connected via convolution filters, represented by dashed, dotted and solid lines. For the sake of simplicity, connections between input-to-output (*IO*) channels are not shown.

In this communication, we aim to produce this type of network by using a stochastic approach that yields random networks with configurable complexity. We are motivated by the fact that network ensembling methods thrive among models with higher variance (Dietterich, 2000 ▸). These sparse mixed-scale networks (SMSNets), illustrated in Fig. 4 ▸, are stochastically configured, both topologically with varying random connections and morphologically with convolutions of different random dilations assigned to each connection. This random nature of model architectures produces additional diversity and higher variance among many models, making them suitable for ensemble methods (Dietterich, 2000 ▸; Ganaie *et al.*, 2022 ▸). Each SMSNet is produced using the following user-specified hyperparameters:

(i) *d*: the number of nodes between the input (*I*) node and the output (*O*) node.

(ii) 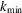 , 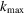 : the global minimum and maximum number of edges per node. By default, these are set to 1 and (*d* + 1), respectively. Adjustments are made on a node level based on their depth.

(iii) *LL*_γ_: the degree distribution parameter. The number of edges *n*_*j*_ at node *j* is a random number drawn from a distribution with density proportional to 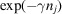 , with 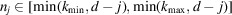 .

(iv) *LL*_α_: the skip-connection distribution parameter governing the probability for an edge to be assigned between node *i* and node *j*, proportional to 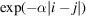 .

(v) *P*_*IL*_: the probability for an edge between input node *I* and any of the intermediate hidden nodes *L*.

(vi) *P*_*LO*_: the probability for an edge between an intermediate hidden node *L* and the output node *O*.

(vii) *P*_*IO*_: a Boolean variable that allows edges between all channels in input node *I* and output node *O*.

DLSIA defaults to the following hyperparameters: *d* = 20, 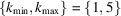 , 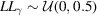 , 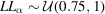 and {*P*_*IL*_, *P*_*LO*_, *P*_*IO*_} = {1, 1, 1}. But when searching parameter space, we recommend first increasing network depth *d* and once again specifying custom dilations of [2^0^, 2^1^, 2^2^,…, 2^*n*^] from which to sample.

Important to note are two observations regarding SMSNets. Firstly, in typical applications with sufficient amounts of labeled data, no individual SMSNet will outperform a more traditional convolutional-based architecture of similar depth *d*. Instead, we typically employ them in multi-network ensembling schemes. Secondly, the exception to this is in applications with limited or incomplete labeled data – individual SMSNets learn a proper segmentation where larger TUNets may completely fail to converge. We demonstrate this phenomenon below in Section 4.2. In this example, TUNets failed to learn a supervised segmentation from sparsely labeled training data. However, we were able to leverage predictions from an ensemble of several low-parameter SMSNets, each with varied architectures generated stochastically and independently using the above hyperparameters available in DLSIA.

## 3. Utility functions and hyperparameter tuning

### 3.1. DLSIA utility functions

In addition to custom CNN architectures, DLSIA offers a number of tools to assist in the end-to-end training process.

(i) Training scripts. DLSIA offers comprehensive training scripts for effortlessly loading data and customizing training instances. Researchers can easily fine-tune a range of essential parameters, including optimizer selection, learning rate, learning schedulers, gradient clipping, early stopping and automatic mixed precision. This flexibility ensures that users can tailor their training process to the unique demands of their scientific image analysis tasks, while efficiently optimizing model performance.

(ii) Custom loss functions. In addition to standard classification loss functions such as the cross-entropy provided by PyTorch, DLSIA provides a collection of custom loss functions designed to tackle specific challenges in scientific image analysis. The Dice loss (Sorensen, 1948 ▸) is an alternative to the cross-entropy loss that measures the overlap between predicted and ground-truth masks. The focal loss (Lin *et al.*, 2017 ▸) aids in handling imbalanced data sets by prioritizing hard-to-classify samples during training. The Tversky loss (Tversky, 1977 ▸) offers a fine-tuned balance between false positives and false negatives, granting users more control over the desired trade-offs during training.

(iii) Random data loaders. In PyTorch, random data splitters are often used for creating separate training, validation and testing data sets from a larger data set, a crucial step in training a robust machine learning model. These tools, such as the RandomSplit function, work by randomly assigning a certain proportion of the data set to each subset. This ensures an unbiased distribution of data points, aiding in preventing overfitting and improving the generalization capability of the model. In essence, random data splitters provide a quick and efficient method to divide data sets, paving the way for effective model training and evaluation processes.

While random data splitters in PyTorch excel in scenarios with large data volumes, their effectiveness can diminish in segmentation problems with a shortage of images. This is because they operate at the image level, meaning they cannot split and shuffle small data sets effectively for robust training and testing. To overcome this limitation, DLSIA introduces random data loaders that perform splitting at a more granular pixel level, creating randomized disjoint sets. This allows for more representative distributions of training and validation data, even in situations with limited images, leading to better model performance and generalizability.

(iv) Conformal estimation methods. DLSIA offers conformal estimation methods (Angelopoulos & Bates, 2021 ▸), enabling researchers to determine confidence intervals for their model predictions. By quantifying uncertainty in predictions, calibrated prediction sets with user-specified coverage are provided, allowing one to make informed decisions in critical applications.

## 4. Applications using DLSIA

We use DLSIA in the following examples to build end-to-end deep learning workflows. Section 4.1 uses MSDNets and tunable U-Nets for inpainting purposes, which are shown to inpaint favorably compared with traditional inpainting algorithms such as biharmonic function approximation. Here, network training was performed on a single 40 GB capacity Nvidia A100 GPU. Additionally, in Sections 4.2 and 4.3 validation of SMSNet ensembling and autoencoder latent space clustering was performed on a single 24 GB memory capacity Nvidia RTX 3090 GPU, along with a 20-thread I9-10900X Intel Core CPU for loading, distributing and receiving work calls to and from the GPU. All training was performed using the ADAM optimizer (Kingma & Ba, 2014 ▸).

### 4.1. Inpainting X-ray scattering images with U-Nets and MSDNets

Image inpainting is a restoration process that estimates the contents of missing regions within images and videos. Several machine learning (ML) approaches exist for inpainting (Elharrouss *et al.*, 2020 ▸; Jam *et al.*, 2020 ▸), chief among them being competing dual-model generative adversarial networks (GANs) (Chen *et al.*, 2021*a* ▸; Zhao *et al.*, 2020 ▸) and partial convolutional operators which augment traditional convolutional layers with adaptive kernel masking (Liu *et al.*, 2018 ▸). While inpainting has recently gained popularity in non-scientific communities for its ability to blindly fill in pictures of heavily masked faces, inpainting in X-ray scattering sciences is limited to only a handful of previous studies which heavily exploit symmetry (Liu *et al.*, 2017 ▸). Since beamline scientists are currently using ML-based algorithms to process the large amount of data they collect (Chen *et al.*, 2021*b* ▸), it is of great importance to reconstruct the missing regions to avoid the introduction of distortion and bias to the post-processing ML analysis.

**Figure 5:**
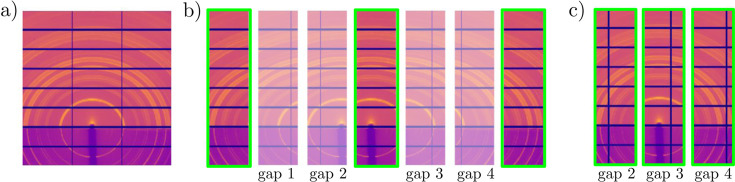
Inpainting data augmentation process to artificially present new vertical gaps with ground-truth information behind them. (*a*) Input data are (*b*) cropped into seven overlapping images, introducing new vertical gaps in one of four positions in the non-highlighted images. (*c*) Highlighted images constitute the original input, but artificial gaps are randomly inserted in one of the four new gap positions.

Hence, DLSIA was employed to inpaint the missing pixel information in vertical and horizontal detector gaps in X-ray scattering data sets. In the study of Chavez *et al.* (2022 ▸), the ground-truth information exists for the missing horizontal gap data which can be used for training, though missing gap data information is entirely nonexistent for the vertical bars. To alleviate this constraint, data augmentation was performed. Outlined in Fig. 5 ▸, this augmentation process artificially introduced vertical bar gaps in new positions which contained ground-truth data behind them.

**Figure 6:**
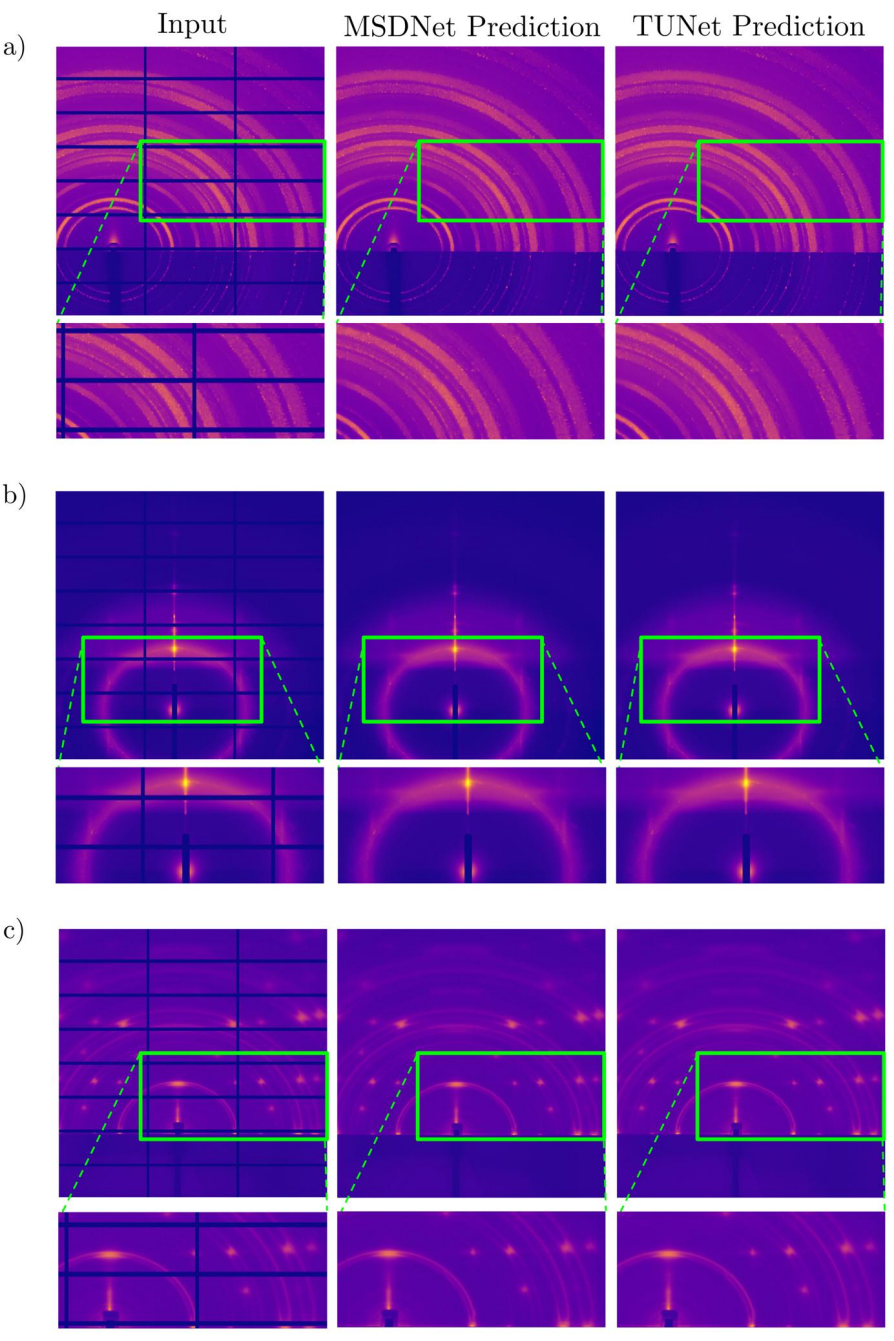
Inpainting of X-ray scattering vertical and horizontal detector gaps using U-Net and MSDNet for (*a*) a grazing-incidence X-ray scattering (GISAXS) pattern of crystalline disordered material, (*b*) a transmission SAXS pattern exhibiting diffuse rings and (*c*) a GISAXS pattern of a crystalline material with a high degree of order.

Two distinct CNNs quite capable at full-image pixel-by-pixel segmentation, a U-Net and an MSDNet, are implemented to see if their capabilities translated to the task of inpainting the gaps. Once the data augmentation steps were complete, nearly 15 000 training images were used, of which three are shown in Fig. 5 ▸(*c*). The *L*_1_ loss metric, which gauges differences between gap predictions and ground truth, was chosen as the target function to minimize. The *L*_2_ loss was also tested but resulted in more blurring, as is consistent with previous inpainting studies (Isola *et al.*, 2017 ▸). Of several different hyperparameter combinations tested, a depth-4 U-Net with ∼8.56 million parameters and a 200-layer MSDNet with ∼0.18 million parameters were the best performing networks, both achieving correlation coefficient scores of >0.998 between predicted gaps and ground truth. The inpainting predictions are displayed in Fig. 6 ▸. While the inpainted gaps do not represent recorded truth and should not be used to derive physical quantities, there is significant evidence in the viability of using gap inpainting for further downstream ML analyses. In particular, the inpainting predictions and their dimensionally reduced autoencoder latent space representations, as compared with non-inpainted and gapped counterparts, are shown to have more favorable compressed representations that can be used for classification or image retrieval purposes (Chavez *et al.*, 2022 ▸).

### 4.2. Detecting 3D fibers in X-ray tomographic reconstructions of concrete using SMSNet ensembling

Fiber reinforcement in concrete plays a fundamental role in enhancing the material’s properties, delivering increased tensile strength, superior shrinkage control, and enhanced flex-induced crack, blast and fire resistances (Beckmann *et al.*, 2021 ▸; Naser *et al.*, 2019 ▸; Zollo, 1997 ▸). As concrete naturally has good compression resistance but lower tensile strength, fibers can be used to improve this tensional weakness, ensuring the material can endure greater tensile stresses. Furthermore, fibers significantly contribute to the concrete’s toughness and durability, providing heightened resistance to impact and abrasion damage (Yuhazri *et al.*, 2020 ▸). Simultaneously, the integral role of fibers in mitigating shrinkage throughout the curing process and the concrete’s lifetime ensures overall enhanced longevity of the structure (Aghaee & Khayat, 2021 ▸).

Understanding the structural distribution of fibers within the concrete matrix is pivotal for comprehending the properties of the composite material and consequently the design of better concrete mixtures. Fiber distribution, orientation and density greatly impact the overall performance of the concrete, influencing its strength, ductility and fracture resistance (Raju *et al.*, 2020 ▸). This characterization can be achieved through techniques such as X-ray tomography, as performed by Wagner & Maas (2023 ▸). Here, the authors use X-ray tomography to produce a volumetric reconstruction of polyethylene fibers distributed in strain-hardened cement composites, commonly used to gauge resistance to cracking under controlled tensile loading (Mechtcherine, 2013 ▸). While the authors use the volumetric reconstruction data set (available to download at https://doi.org/10.34740/KAGGLE/DS/2894881) supplemented with extensive augmentation to validate a number of 3D segmentation models, we instead perform 2D manual binary segmentation with much more incomplete and sparsely curated ground-truth data. This low data constraint allows us to test the feasibility of training SMSNets against limited ground-truth data and discuss their advantages over U-Nets in these data-limited regimes.

**Figure 7:**
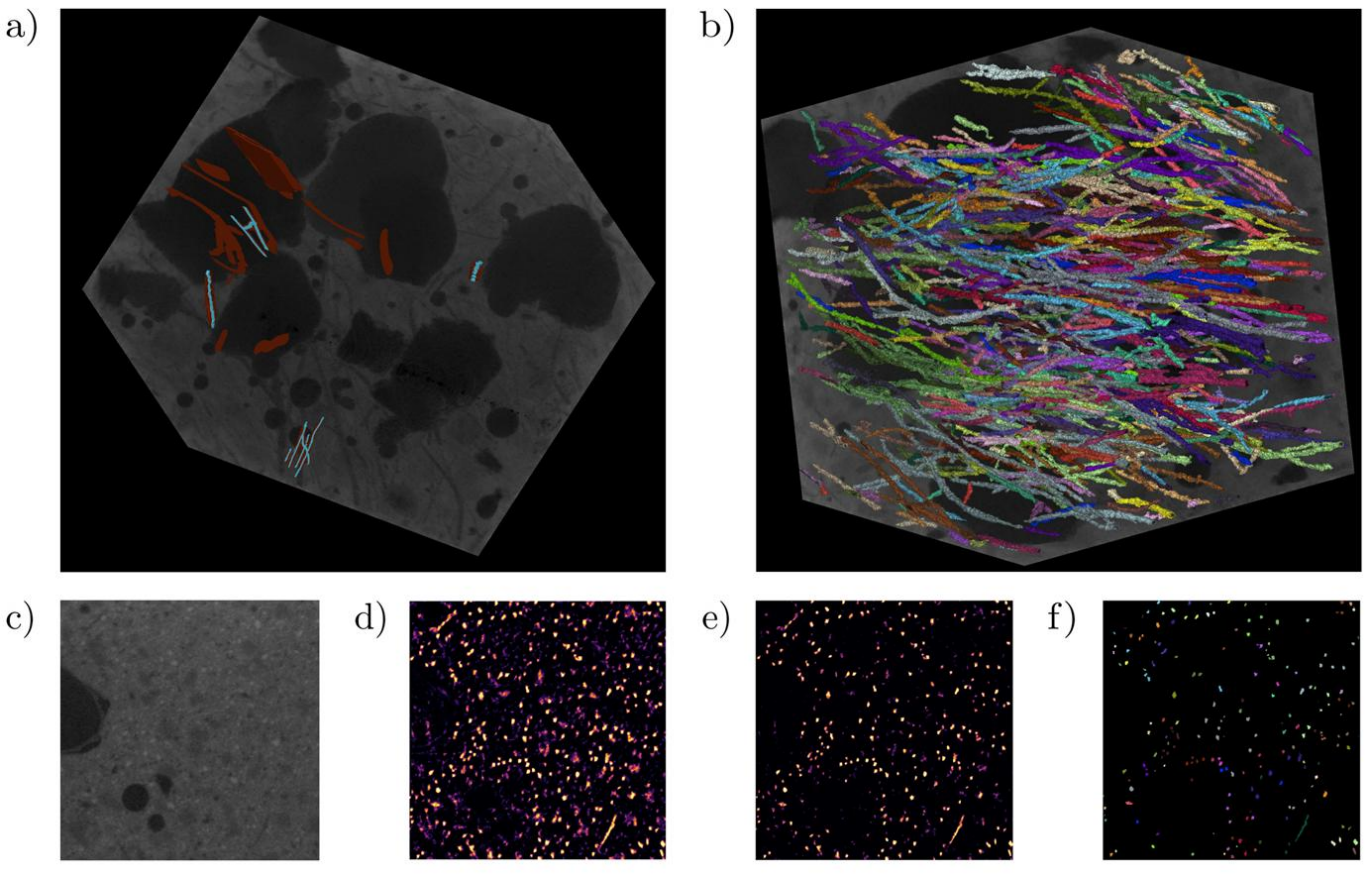
Ensemble network predictions of fibers in concrete. (*a*) Sparse binary labeling of target fibers (cyan) and background (brown). (*b*) Aggregated network predictions. (*c*) Cross-sectional slice of raw training data. (*d*) Probability map of aggregated network predictions. (*e*) Probability map with standard deviations subtracted. (*f*) Cross-sectional view of instance segmented fibers derived from (*e*).

The limited ground-truth data curation consisted of manual segmentation using the *Napari* software (Sofroniew *et al.*, 2022 ▸) resulting in the sparse and incomplete hand-annotation of only six fibers, consisting of ∼245 000 labeled pixels with a 10:2 background-to-foreground ratio. Hand-annotations used for training are displayed in Fig. 7 ▸(*a*). This selection was restricted to a few locations with the focus of balancing accuracy – particularly when labeling the border between classes – and overall speed of annotation to maintain a manageable workload.

The prepared data were then subjected to (i) an ensemble of five DLSIA-instantiated SMSNets, each with a different stochastically generated architecture and approximately 45 000 parameters, and (ii) several different sizes of TUNets ranging over two orders of magnitude in learnable weights. Each TUNet failed to produce a meaningful segmentation model, likely due to the sparsity of the labels. The SMSNets here proved to be more resilient in low-data regimes, in line with previous observations (Pelt & Sethian, 2018 ▸). Additionally, we note that each individual SMSNet instance has a stochastically generated architecture, thus simplifying the hyperparameter fine-tuning process.

**Figure 8:**
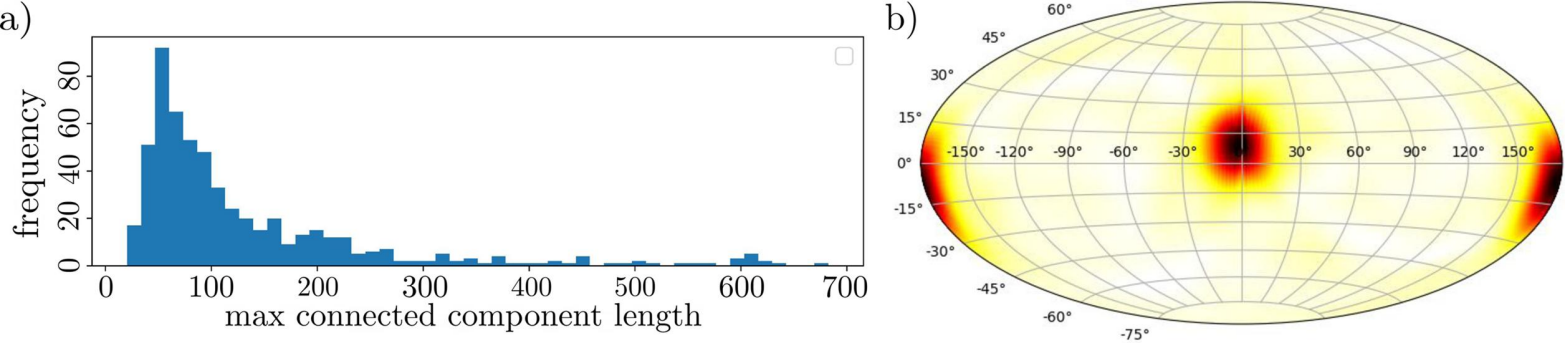
Summary statistics of fiber segmentation predictions. Displayed are (*a*) a histogram plot of fiber lengths and (*b*) an equal-area Hammer projection of the autocorrelation function of the 3D segmentation results at a radius of 30 pixels from the origin, showing a general anisotropic distribution of the direction of the fibers.

The SMSNet multi-network mean prediction probabilities are displayed in Fig. 7 ▸(*d*). However, we choose to leverage the multi-network standard deviation and keep only those pixel predictions whose probability remains over 50% after subtracting a single standard deviation, pictured in Fig. 7 ▸(*e*). A subsequent analysis using the external Python package *cc3d* (Wu *et al.*, 2021 ▸) involved 3D instance segmentation using a decision tree augmented 3D variant of connected components (Wu *et al.*, 2005 ▸). Additionally, *cc3d* allowed for the removal of small connected components – a so-called ‘dusting’ – below some user-defined threshold. Both a histogram of the end-to-end length of the instance segmented fibers and a Hammer–Aitoff projection (Tobler, 1964 ▸) of the surface of an origin-centered 30-pixel sphere of the autocorrelation function of the segmented labels – essentially measuring the directional distribution of the segmented fibers – are shown in Fig. 8 ▸, providing critical insights into the morphology and organization of the segmented fibers that can be used to understand, predict or design properties of fiber-reinforced concrete.

### 4.3. Autoencoder compression and latent space clustering

We present the results of our clustering approach on the highly compressed autoencoder latent space using synthetic data consisting of 64 × 64 tiles, each containing one of four random shapes (circle, triangle, rectangle and annulus) that are randomly sized and rotated by a random degree around their centers. We applied a four-layer 16-base channel autoencoder that bottlenecks to a 16 × 1 sized latent space (or feature space) to reconstruct the input data, optimized on the mean square error loss. To assess the quality of our model reconstruction, we found the Pearson cross-correlation scored against the original images, which yielded an impressive score of approximately 0.98.

**Figure 9:**
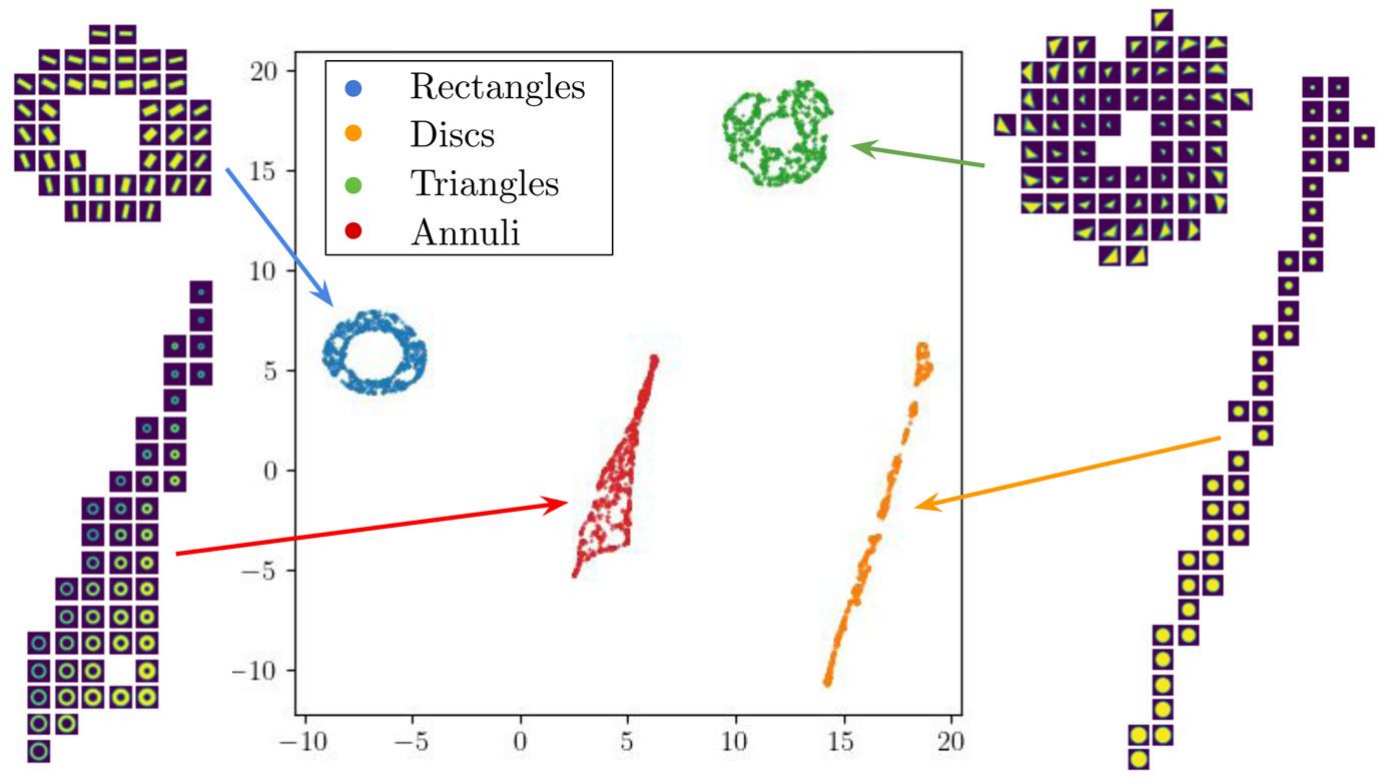
Autoencoder latent space representation, further compressed by *U-Map*, of randomly sized and oriented shapes.

Once the model was sufficiently trained, we passed new images through the trained autoencoder to obtain their 16 × 1 latent space representation, a 256-factor compression of the data. To visualize and analyze the clustering behavior, we further compressed the latent space down to two real numbers using *U-Map* (McInnes *et al.*, 2018 ▸), allowing us to generate meaningful scatter plots in Cartesian coordinates. As illustrated in Fig. 9 ▸, our approach exhibits clear, distinct clustering results between each of the four shapes. Moreover, the approach handles the variations in shape orientation and size remarkably well, with clear transitions between each shape’s size and orientation within each cluster.

## 5. Discussion and conclusions

We introduce DLSIA (Deep Learning for Scientific Image Analysis), a Python-based deep learning convolutional neural network library aimed at bringing a new level of user-customizability to researchers and their image analysis tasks. Offering simplified network construction, multiple proven network architectures and an array of tunable training parameters, DLSIA provides a versatile platform allowing users to explore diverse network settings. DLSIA-instantiated networks and workflows were validated through three separate applications: (i) semantic segmentation of fibers in X-ray tomographic reconstruction of concrete data using an ensemble of SMSNets, (ii) inpainting of missing gap information in X-ray scattering data using U-Nets and MSDNets, and (iii) investigation into clustering autoencoder latent space on synthetic shape data.

The above algorithms are implemented in a set of Python3 routines, and are *pip* installable (via pip install dlsia). Additionally, some DLSIA modules for custom MSDNet, autoencoder and U-Net instantiation for segmentation purposes are available within the MLExchange collaborative machine learning platform for facility scientists (Zhao *et al.*, 2022 ▸; Hao *et al.*, 2023 ▸; Hexemer *et al.*, 2021 ▸). Trained networks and sample notebooks for examples listed in Sections 4.1 and 4.2 can be found online at https://huggingface.co/phzwart/dlsia_inpainting_saxs_gisaxs and https://huggingface.co/phzwart/dlsia_concrete_fiber, respectively.

## Figures and Tables

**Table 1:** DLSIA utility modules and functions

Script/module	Description
baggins	Contains ensembling-based methods for combining neural networks
conformalize_segmentation	Used to perform conformal estimation on a set of model predictions
custom_losses	Contains an array of popular loss functions suitable for image segmentation
draw_sparse_network	Visualizes the topology and layout of individual SMSNets
helpers	Contains several minor utility functions, including functions for retrieving the current computing device, counting model parameters and convolutional filters, and initiating PyTorch DataLoader classes
latent_space_viewer	Visualizes images in autoencoder latent space upon a single instance of U-Map, as viewed in Fig. 9 ▸
msae	Creates autoencoder networks; mixed-scale functionality is forthcoming
msdnet	Creates mixed-scale dense networks (MSDNets)
plots	Contains a suite of plotting tools for model segmentation, regression and aggregation
random_shapes	Generates random circles, rectangles, triangles and annuli used in Section 4.3 with random size, orientation and user-defined Gaussian noise
randomized_data_loader	Returns input data into random partition of training and testing data
scale_up_down	Contains modules for data resizing used in TUNets, U-Nets and autoencoders
segmentation_metrics	Computes F1 scores for evaluating quality of model segmentation performance
smsnet	Creates random, sparse mixed-scale networks (SMSNets) for 2D data
smsnet3d	Creates random SMSNets for 3D data
train_scripts	Contains end-to-end model training procedures and evaluation metrics for segmentation and regression problems
tunet	Creates custom, tunable U-Nets
tunet3plus	Creates U-Net3+, a new variant of the classic U-Net featuring dense skip-connection aggregating features from all network layers

## Appendix A DLSIA modules and subroutines

The DLSIA library contains many subroutines and functionalities not listed or mentioned in the text above. Table 1 ▸ references a bulk of the DLSIA modules available for use. For full documentation and listing of modules, please see https://dlsia.readthedocs.io/en/latest/.

## References

[bb1] Aghaee, K. & Khayat, K. H. (2021). *Constr. Build. Mater.* **305**, 124586.

[bb2] Angelopoulos, A. N. & Bates, S. (2021). *arXiv*:2107.07511.

[bb3] Beckmann, B., Bielak, J., Bosbach, S., Scheerer, S., Schmidt, C., Hegger, J. & Curbach, M. (2021). *Civ. Eng. Des.* **3**, 99–109.

[bb4] Berral, J. L., Aranda, O., Dominguez, J. L. & Torres, J. (2021). *arXiv*:2110.15884.

[bb5] Blalock, D., Ortiz, J. J. G., Frankle, J. & Guttag, J. (2020). *arXiv*:2003.03033.

[bb6] Chavez, T., Roberts, E. J., Zwart, P. H. & Hexemer, A. (2022). *J. Appl. Cryst.* **55**, 1277–1288. 10.1107/S1600576722007105 PMC9533742 36249508

[bb7] Chen, Y., Zhang, H., Liu, L., Chen, X., Zhang, Q., Yang, K., Xia, R. & Xie, J. (2021*a*). *Appl. Intell.* **51**, 3460–3474.

[bb8] Chen, Z., Andrejevic, N., Drucker, N. C., Nguyen, T., Xian, R. P., Smidt, T., Wang, Y., Ernstorfer, R., Tennant, D. A., Chan, M. & Li, M. (2021*b*). *Chem. Phys. Rev.* **2**, 031301.

[bb9] Deyhle, H., White, S. N., Botta, L., Liebi, M., Guizar-Sicairos, M., Bunk, O. & Müller, B. (2018). *J. Imaging*, **4**, 81.

[bb10] Dietterich, T. G. (2000). *International Workshop On Multiple Classifier Systems*, pp. 1–15. Berlin, Heidelberg: Springer.

[bb11] Douarre, C., Schielein, R., Frindel, C., Gerth, S. & Rousseau, D. (2018). *J. Imaging*, **4**, 65.

[bb12] Drozdzal, M., Vorontsov, E., Chartrand, G., Kadoury, S. & Pal, C. (2016). *Deep Learning and Data Labeling for Medical Applications*, pp. 179–187. Cham: Springer.

[bb13] Elharrouss, O., Almaadeed, N., Al-Maadeed, S. & Akbari, Y. (2020). *Neural Process. Lett.* **51**, 2007–2028.

[bb14] Finzi, M., Stanton, S., Izmailov, P. & Wilson, A. G. (2020). *Proc. Mach. Learning Res.* **119**, 3165–3176.

[bb15] Ganaie, M. A., Hu, M., Malik, A., Tanveer, M. & Suganthan, P. (2022). *Eng. Appl. Artif. Intell.* **115**, 105151.

[bb16] Gong, K., Berg, E., Cherry, S. R. & Qi, J. (2020). *Proc. IEEE*, **108**, 51–68. 10.1109/JPROC.2019.2936809 PMC10691821 38045770

[bb17] Hao, G., Roberts, E. J., Chavez, T., Zhao, Z., Holman, E. A., Yanxon, H., Green, A., Krishnan, H., Ushizima, D., McReynolds, D., Schwarz, N., Zwart, P. H., Hexemer, A. & Parkinson, D. (2023). *IS&T Int. Symp. Electron Imaging*, **35**, IPAS-290. 10.2352/ei.2023.35.9.ipas-290 PMC10735246 38130938

[bb18] Hexemer, A., Zwart, P. H., McReynolds, D., Green, A. & Chavez Esparza, T. A. (2021). *MLExchange v1.* Technical Report. Lawrence Berkeley National Laboratory (LBNL), Berkeley, CA, USA.

[bb19] Ioffe, S. & Szegedy, C. (2015). *Proc. Mach. Learning Res*, **37**, 448–456.

[bb20] Isola, P., Zhu, J.-Y., Zhou, T. & Efros, A. A. (2017). *2017 IEEE Conference on Computer Vision and Pattern Recognition*, pp. 5967–5976. https://doi.org/10.1109/CVPR.2017.632.

[bb21] Jam, J., Kendrick, C., Walker, K., Drouard, V., Hsu, J. G.-S. & Yap, M. H. (2020). *Comput. Vis. Image Underst.* p. 103147.

[bb22] Jung, C. & Kim, C. (2014). *Cytometry Pt A*, **85**, 709–718. 10.1002/cyto.a.22467 24677732

[bb23] Kaur, P., Singh, G. & Kaur, P. (2018). *Curr. Med. Imaging*, **14**, 675–685. 10.2174/1573405613666170428154156 PMC6225344 30532667

[bb24] Kiapour, M. H., Yager, K., Berg, A. C. & Berg, T. L. (2014). *IEEE Winter Conference on Applications of Computer Vision (WACV)*, pp. 933–940.

[bb25] Kingma, D. P. & Ba, J. (2014). *arXiv*:1412.6980.

[bb26] Kinnison, J., Kremer-Herman, N., Thain, D. & Scheirer, W. (2018). *IEEE Winter Conference on Applications of Computer Vision (WACV)*, pp. 738–747.

[bb27] Kirman, J., Johnston, A., Kuntz, D. A., Askerka, M., Gao, Y., Todorović, P., Ma, D., Privé, G. G. & Sargent, E. H. (2020). *Matter*, **2**, 938–947.

[bb28] Kromp, F., Bozsaky, E., Rifatbegovic, F., Fischer, L., Ambros, M., Berneder, M., Weiss, T., Lazic, D., Dörr, W., Hanbury, A., Beiske, K., Ambros, P. F., Ambros, I. M. & Taschner-Mandl, S. (2020). *Sci. Data*, **7**, 262. 10.1038/s41597-020-00608-w PMC7419523 32782410

[bb29] Kumar, P., Nagar, P., Arora, C. & Gupta, A. (2018). *25th IEEE International Conference on Image Processing (ICIP)*, pp. 3503–3507.

[bb30] Lawhern, V. J., Solon, A. J., Waytowich, N. R., Gordon, S. M., Hung, C. P. & Lance, B. J. (2018). *J. Neural Eng.* **15**, 056013. 10.1088/1741-2552/aace8c 29932424

[bb31] LeCun, Y., Bottou, L., Bengio, Y. & Haffner, P. (1998). *Proc. IEEE*, **86**, 2278–2324.

[bb32] Li, Y., Chouzenoux, E., Charmettant, B., Benatsou, B., Lamarque, J.-P. & Lassau, N. (2021). *IEEE 18th International Symposium on Biomedical Imaging (ISBI)*, pp. 611–615.

[bb33] LiKamWa, R., Hou, Y., Gao, J., Polansky, M. & Zhong, L. (2016). *ACM SIGARCH Comput. Arch. News*, **44**, 255–266.

[bb34] Lin, T.-Y., Goyal, P., Girshick, R., He, K. & Dollár, P. (2017). *Proceedings of the IEEE International Conference on Computer Vision*, pp. 2980–2988.

[bb35] Liu, G., Reda, F. A., Shih, K. J., Wang, T.-C., Tao, A. & Catanzaro, B. (2018). *Proceedings of the European Conference on Computer Vision (ECCV)*, pp. 85–100.

[bb36] Liu, J., Lhermitte, J., Tian, Y., Zhang, Z., Yu, D. & Yager, K. G. (2017). *IUCrJ*, **4**, 455–465. 10.1107/S2052252517006212 PMC5571808 28875032

[bb37] Liu, S., Melton, C. N., Venkatakrishnan, S., Pandolfi, R. J., Freychet, G., Kumar, D., Tang, H., Hexemer, A. & Ushizima, D. M. (2019). *MRS Commun.* **9**, 586–592.

[bb38] Manifold, B., Thomas, E., Francis, A. T., Hill, A. H. & Fu, D. (2019). *Biomed. Opt. Expr.* **10**, 3860–3874. 10.1364/BOE.10.003860 PMC6701518 31452980

[bb39] McInnes, L., Healy, J. & Melville, J. (2018). *arXiv*:1802.03426.

[bb40] Mechtcherine, V. (2013). *Constr. Build. Mater.* **41**, 365–373.

[bb41] Naser, M., Hawileh, R. & Abdalla, J. (2019). *Eng. Struct.* **198**, 109542.

[bb42] Noh, K. J., Park, S. J. & Lee, S. (2019). *Comput. Methods Programs Biomed.* **178**, 237–246. 10.1016/j.cmpb.2019.06.030 31416552

[bb43] Park, J., Li, S., Wen, W., Tang, P. T. P., Li, H., Chen, Y. & Dubey, P. (2016). *arXiv*:1608.01409.

[bb44] Pelt, D. M., Batenburg, K. J. & Sethian, J. A. (2018). *J. Imaging*, **4**, 128.

[bb45] Pelt, D. M. & Sethian, J. A. (2018). *Proc. Natl Acad. Sci. USA*, **115**, 254–259. 10.1073/pnas.1715832114 PMC5777062 29279403

[bb46] Radivojević, T., Costello, Z., Workman, K. & Martin, H. G. (2020). *Nat. Commun.* **11**, 1–14. 10.1038/s41467-020-18008-4 PMC7519645 32978379

[bb47] Raju, R. A., Lim, S., Akiyama, M. & Kageyama, T. (2020). *Constr. Build. Mater.* **262**, 119963.

[bb48] Ronneberger, O., Fischer, P. & Brox, T. (2015). *International Conference on Medical Image Computing and Computer-Assisted Intervention*, pp. 234–241. Munich: Springer.

[bb49] Rumelhart, D. E., Hinton, G. E. & Williams, R. J. (1985). *Learning Internal Representations by Error Propagation.* Technical Report. California University San Diego, La Jolla Institute for Cognitive Science, La Jolla, CA, USA.

[bb50] Schirrmeister, R. T., Springenberg, J. T., Fiederer, L. D. J., Glasstetter, M., Eggensperger, K., Tangermann, M., Hutter, F., Burgard, W. & Ball, T. (2017). *Hum. Brain Mapp.* **38**, 5391–5420. 10.1002/hbm.23730 PMC5655781 28782865

[bb51] Schoonhoven, R., Hendriksen, A. A., Pelt, D. M. & Batenburg, K. J. (2020). *arXiv*:2011.06923.

[bb53] Sofroniew, N., Lambert, T., Evans, K., Nunez-Iglesias, J., Bokota, G., Winston, P., Peña-Castellanos, G., Yamauchi, K., Bussonnier, M., Doncila Pop, D., Can Solak, A., Liu, Z., Wadhwa, P., Burt, A., Buckley, G., Sweet, A., Migas, L., Hilsenstein, V., Gaifas, L., Bragantini, J., Rodriguez-Guerra, J., Munoz, H., Freeman, J., Boone, P., Lowe, A., Gohlke, C., Royer, L., Pierre, A., Har-Gil, H. & McGovern, A. (2022). *napari: a Multi-Dimensional Image Viewer for Python (v0.4.17rc8)*, https://doi.org/10.5281/zenodo.7276432.

[bb54] Sorensen, T. (1948). *Biol. Skrifter*, **5**, 1–34.

[bb55] Springenberg, J. T., Dosovitskiy, A., Brox, T. & Riedmiller, M. (2014). *arXiv*:1412.6806.

[bb56] Sun, S., Hartono, N. T. P., Ren, Z. D., Oviedo, F., Buscemi, A. M., Layurova, M., Chen, D. X., Ogunfunmi, T., Thapa, J., Ramasamy, S., Settens, C., DeCost, B. L., Kusne, A. G., Liu, Z., Tian, S. I. P., Peters, I. M., Correa-Baena, J. & Buonassisi, T. (2019). *Joule*, **3**, 1437–1451.

[bb57] Tabar, Y. R. & Halici, U. (2016). *J. Neural Eng.* **14**, 016003. 10.1088/1741-2560/14/1/016003 27900952

[bb58] Tobler, W. (1964). *Surv. Rev.* **17**, 240–243.

[bb59] Tong, T., Li, G., Liu, X. & Gao, Q. (2017). *Proceedings of the IEEE International Conference on Computer Vision*, pp. 4799–4807.

[bb60] Tversky, A. (1977). *Psychol. Rev.* **84**, 327–352.

[bb61] Wagner, F. & Maas, H.-G. (2023). *Int. Arch. Photogramm. Remote Sens. Spat. Inf. Sci. XLVIII-1/W2-2023*, pp. 1667–1676.

[bb62] Wäldchen, J. & Mäder, P. (2018). *Methods Ecol. Evol.* **9**, 2216–2225.

[bb63] Wang, B., Yager, K., Yu, D. & Hoai, M. (2017). *IEEE Winter Conference on Applications of Computer Vision (WACV)*, pp. 697–704.

[bb64] Wang, H., Qin, C., Zhang, Y. & Fu, Y. (2021). *arXiv*:2103.06460.

[bb52] Wu, J., Silversmith, W. M., Lee, K. & Seung, H. S. (2021). *Nat. Methods*, **18**, 328–330. 10.1038/s41592-021-01088-5 33750934

[bb65] Wu, K., Otoo, E. & Suzuki, K. (2005). *Two Strategies to Speed Up Connected Component Labeling Algorithms.* Technical Report. Lawrence Berkeley National Laboratory (LBNL), Berkeley, CA, USA.

[bb66] Xing, F., Xie, Y., Su, H., Liu, F. & Yang, L. (2017). *IEEE Trans. Neural Networks Learning Systems*, **29**, 4550–4568. 10.1109/TNNLS.2017.2766168 29989994

[bb67] Yu, F. & Koltun, V. (2015). *arXiv*:1511.07122.

[bb68] Yuhazri, M., Zulfikar, A. & Ginting, A. (2020). *IOP Conf. Ser. Mater. Sci. Eng.* **1003**, 012135.

[bb69] Zhao, L., Mo, Q., Lin, S., Wang, Z., Zuo, Z., Chen, H., Xing, W. & Lu, D. (2020). *Proceedings of the IEEE/CVF Conference on Computer Vision and Pattern Recognition (CVPR).* pp. 5741–5750. https://doi.org/10.1109/CVPR42600.2020.00578.

[bb70] Zhao, Z., Chavez, T., Holman, E. A., Hao, G., Green, A., Krishnan, H., McReynolds, D., Pandolfi, R. J., Roberts, E. J., Zwart, P. H., Yanxon, H., Schwarz, N., Sankaranarayanan, S., Kalinin, S. V., Mehta, A., Campbell, S. I. & Hexemer, A. (2022). *4th Annual Workshop on Extreme-Scale Experiment-in-the-Loop Computing (XLOOP)*, pp. 10–15. IEEE. 10.1109/xloop56614.2022.00007 PMC10733127 38131031

[bb71] Zhou, Z., Siddiquee, M. M. R., Tajbakhsh, N. & Liang, J. (2018). *Deep Learning in Medical Image Analysis and Multimodal Learning for Clinical Decision Support*, pp. 3–11. Granada: Springer. 10.1007/978-3-030-00889-5_1 PMC7329239 32613207

[bb72] Zhou, Z., Siddiquee, M. M. R., Tajbakhsh, N. & Liang, J. (2020). *IEEE Trans. Med. Imaging*, **39**, 1856–1867. 10.1109/TMI.2019.2959609 PMC7357299 31841402

[bb73] Ziletti, A., Kumar, D., Scheffler, M. & Ghiringhelli, L. M. (2018). *Nat. Commun.* **9**, 2775. 10.1038/s41467-018-05169-6 PMC6050314 30018362

[bb74] Zollo, R. F. (1997). *Cem. Concr. Compos.* **19**, 107–122.

